# LoRaWAN for Smart Campus: Deployment and Long-Term Operation Analysis

**DOI:** 10.3390/s20236721

**Published:** 2020-11-24

**Authors:** Rumana Yasmin, Konstantin Mikhaylov, Ari Pouttu

**Affiliations:** Centre for Wireless Communications, University of Oulu, P.O. Box 4500, 90014 Oulu, Finland; konstantin.mikhaylov@oulu.fi (K.M.); ari.pouttu@oulu.fi (A.P.)

**Keywords:** LoRaWAN, IoT, smart campus, experiment, testbed, empirical, analysis, packet error rate, losses, interference

## Abstract

The recent years have gradually increased the value of wireless connectivity, making it the de facto commodity for both human users and the machines. In this paper, we summarize our experiences of deploying and managing for over two years the extensive indoor sensor network composed of more than three hundred devices connected over LoRaWAN low power wide area network (LPWAN) technology. We start by detailing the background and methodology of our deployment and then present the results of analyzing the network’s operation over a period of two years, focusing specifically on identifying the reasons after the packet losses. Our results reveal that despite the common assumptions, in a real-life network, the packets are lost not only during the on-air transmission but also within the backbone. Among the other interesting findings are the observed nonuniform distribution of the packet transmissions by the nodes in the networks, the seasonal effects on the packet delivery, and the observed effects of the interferences on network performance. The empirical results presented in the paper provide valuable insight into the performance of a real-life extensive LoRaWAN network deployed in an indoor environment and thus may be of interest both to the practitioners and academics.

## 1. Introduction

The 21st century has witnessed the internet of things (IoT) advancements delivering a digital revolution for almost every aspect of human life. The Smart Cities are among the best illustrations of these. Among the numerous recently developed Smart City applications are the office building control and monitoring systems [1], patient monitoring system in hospitals [2], IoT-based smart laboratory [3], IoT-based smart edge system for remote health monitoring [4], and smart energy-efficient home automation [5]. In this context, monitoring use cases in real estate, which we use as an umbrella for the versatile monitoring applications operating primarily indoor and close-to-indoor (parking, underground passages, etc.) in a Smart City, are especially perspective and offer many new innovation possibilities [6]. In this paper, we focus on a specific, albeit rather illustrative real-estate concept and scenario—a smart campus. Importantly, a smart campus can become a “seed” for a smart city by allowing students, faculty members, university staff, service providers, and visitors to experiment and experience innovative applications, services, and techniques, thus facilitating and driving the innovation further.

The campuses of the future are increasingly digitized; hyper-connectivity will span out over the entire premises, emphasizing the invention of the novel applications and use cases. At this time, many use cases, including real estate monitoring, create the need for a diverse landscape of wireless technologies to serve the versatile needs of the diverse IoT applications. In this context, the low power consumption and comprehensive coverage is a key value proposition relevant for many use cases in a real estate application [7,8]. Some of these can be addressed through the use of such existing technologies as Bluetooth or ZigBee, which are typically employed for short-distance communication [9]. However, as a rule of thumb, most of the use cases in real estate require medium- or long-distance communications but feature limited throughput and infrequent communication. To address these needs, the low power wide area network (LPWAN) [7] technologies have been introduced. Even though the typical LPWAN follows a basic star network topology and a single-hop communication model, a single gateway is enough to enable a large number of nondemanding connections. At the same time, communication distances between the end-devices and the gateway can be extended from hundreds of meters up to several kilometers. This makes the LPWAN technologies well-suited in terms of cost effectiveness and deployment ease for serving real estate applications.

The landscape of the LPWAN technologies available today is not uniform, comprising more than 20 different candidate technologies. The three technologies, which are currently being massively rolled out commercially are SigFox [10], NB-IoT [11], and LoRaWAN [12]. However, the SigFox is a closed solution, which is run and developed by one single company. Due to this reason, (i) the information about the details of the SigFox technology in open access is somewhat scarce, and (ii) the closed nature and unavailability of the respective hardware solutions on the market makes this complex for the research community to experiment, upgrade, and test the potential improvements for SigFox [13] technology. Notably, SigFox limits the uplink payload length to only 12 bytes, which complicates its use for various IoT applications offering more data for transfer [14,15]. Due to its closed nature and the limited commercial availability of the elements of infrastructure, as well as limitation of the transmission rate (standard allows up to 140 messages per day [14]), the SigFox technology is hardly reasonable for indoor small-scale private deployments [16] and for the use cases that demand high reliability constraints [17]. The NB-IoT has been available for a while, and the activities taking place with this technology are in process. In addition, NB-IoT mostly relies on telecom operators offering the service and spectrum, i.e., the availability of network coverage and infrastructure. At the same time, LoRaWAN is one of the most widely used wireless technology to connect the ever increasing IoT devices [7,8] over the license-free ISM bands. As we see from the prior studies [18], seamless connectivity from both public (i.e., operator controlled) and a private (i.e., user controlled) network can be enabled by the LoRaWAN technology.

Aside from enabling seamless connectivity, LoRaWAN may bring several potential benefits in IoT deployments for real estate. To get a better understanding of the status of LoRaWAN technology in real estate and the related verticals, we briefly discuss the illustrative results of the relevant previous studies. First of all, the health sector, being one of the imminent and most demanding verticals, requires remote a healthcare monitoring solution to monitor the patients in hospitals and even their own accommodations [19,20]. A biotelemetry monitoring service based on LoRaWAN was implemented and presented in [21]. The system is deployed over a private network with the possibility of error-free transmissions over a long distance (1.1–6 km). Another relevant use case was developed in the context of remote monitoring to manage electricity consumption in an efficient way [22]. Apart from keeping track of household utilities, the system is capable of predicting electricity consumption of a random building in a neighborhood on a particular day. The third use case is related to monitoring energy grids. Fading is always a problem in wireless communications when the signals are transmitted in an urban or a suburban environment. In such an environment, the transmitted signals can be reflected, refracted, or scattered due to buildings, mountains, water, etc., causing fading. LoRaWAN, owing to its fading resilience and nonline-of-sight communication capability, allows for developing a real-time power distribution monitoring system in a suburban area [23,24]. The fourth use case is related to industry. In order to connect many devices with a single gateway, LoRaWAN was leveraged to realize a monitoring system for flower preservation trolleys [25]. The number of sensors attached to the trolleys and connected to a single gateway was 6000. Finally, LoRaWAN was also used in mobility and on-wheels scenarios in an indoor and outdoor environment, respectively [26]. For instance, a wearable device can keep track of fitness parameters such as heartbeat and blood pressure when users are moving. Developing a monitoring system connected with such wearable devices is often challenging. However, LoRaWAN, due to its capability to mitigate Doppler effect and eliminate frequency offset between transmitter and receiver [27], can be suitable for such use cases. From all the mentioned research activities and developments discussed above, it appears that LoRaWAN is a feasible technology to address the needs of versatile real estate use cases. With this aspect, we intended to utilize LoRaWAN to deploy real-life monitoring systems in a large and congested indoor area to make this real estate environment digitized and smart at the university campus.

In order to enable the LoRaWAN in indoor premises and gather information on the real estate environment to offer innovative applications, 331 sensor nodes were deployed at the University of Oulu. The deployed sensor network comprises 1655 different sensors monitoring various environmental parameters such as temperature, humidity, CO_2_ levels, light intensity, and motions in their close proximity. The network is seen as a platform to enable the development of diverse applications. To give one example, among these is the augmented reality-based mobile application visualizing the real-time information of sensor nodes for maintenance purposes and to facilitate selection and booking of the premises for teaching activities and self-study [28].

The primary contribution of this paper is the reported results and the lessons learned while deploying, configuring, utilizing, and monitoring the behavior of this extensive network over a period of about two years. Importantly, what makes our deployment stand out from the mass of commercial deployments is its indoor nature. Specifically, in the paper, we focus on investigating the packet delivery rate and the main reasons for after the loss of the packets in the deployed network, which were observed during the initial analysis, as reported in [29]. In this paper, we extend our analysis both in-time and in-depth to capture the effects present in practical LoRaWAN deployment. The secondary contribution of this manuscript is the systematic deployment methodology summarized in this paper. Overall, the results presented may be of interest to a broad range of audience, including both the practitioners, who plan or already work on the deployment of a large-scale LoRaWAN network, and academics, for which the results presented highlight some of the challenges and issues present in a real-life network.

The manuscript is composed of five sections. Section 2 delivers a detailed introduction and overview of LoRaWAN. Section 3 describes our methodology of deploying the real estate monitoring system and summarizes the results of analyses done prior to deployment. Section 3 also reveals the hardware used, the network setup, and configurations. The experimental results emphasizing several aspects of the deployed network are presented in Section 4. Finally, Section 5 concludes the paper, discusses the key results and lists some of the future activities planned.

## 2. LoRaWAN Technology at a Glance

The LoRaWAN initial standard was developed by the LoRa Alliance and released in 2015 [30]. Afterwards, the first commercial LoRaWAN rolled out in 2016. Currently, a range of mobile network operators and different sensor vendors around the globe have developed commercial solutions leveraging the LoRaWAN technology: Kerlink [31], Multitech [32], KPN [33], Orange [34], Tekelek [35], SK Telecom [36], to name a few. For enabling IoT services, irrespective of the location of end-users, roaming is an aspect considered recently as a joint effort by Orange and KPN [37]. This advancement makes a decisive step towards enabling the cross-continent mobility of LoRaWAN devices. Further, in this section, we briefly overview the key aspects of LoRaWAN technology.

### 2.1. The Physical Layer

The LoRaWAN specification describes several technical aspects specific to the physical-to-media access control (MAC) layer. The releases following the initial one brought updates of the allocated backend interfaces [38] and frequency allocations for different regions [39]. Note that various sub-GHz bands have been assigned for ISM use in the different regions, i.e., 915–928 MHz for Australia, 779–787 MHz for China, 920–923 MHz for South Korea, and 902–928 MHz for the United States. In this paper, the LoRaWAN system is operating in the 868 MHz EU band, unless stated otherwise.

Considering the operation of LoRaWAN in the EU868 MHz ISM band, the two different modulation techniques can be used: LoRa modulation and frequency-shift keying (FSK). The LoRa modulation is utilized more frequently and relies on the chirp spread spectrum (CSS) technique. In this technique, the chirp signal utilizes wideband linear modulated pulses whose frequency varies in order to cover the entire available bandwidth (BW). The use of LoRa modulation provides the possibility to select from a range of spreading factors (SFs). This possibility enables achieving a trade-off between the on-air time and communication range. The robustness in transmission may be increased by adding forward error correction (FEC). The on-air time $T_{OA}$ can be defined as [40]:

(1) $$T_{OA}=\left(\frac{2^{SF}\times 12.25}{BW}\right)+\left\{8+max\left(ceil\left(\frac{8PL-4SF+28+16CRC-20IH}{4\left(SF-2DE\right)}\right)\left(CR+4\right),0\right)\right\},$$

In Equation (1), CRC is defined as 1, IH value is 0 for the uplink transmission, and CR is set to 1 (thus corresponding to the coding rate of 4/5), as dictated by the LoRaWAN specification. DE is obligatory for SF11 and SF12 to enable low data rate optimization using the value of DE as one and is not used for other SFs. For transmitting the data in the uplink, LoRaWAN uses a variant of Aloha channel access, discussed in more detail in the following subsection, with a random selection of a frequency channel to be used. The EU868 MHz end-devices use three frequency channels by default, i.e., 868.1, 868.3, and 868.5 MHz. In these channels, it is possible to use the SF from 7 to 12 with the channel BW 125 kHz. In addition, extra channels can be enabled. Note that the LoRa signals encoded with the different SFs are quasiorthogonal [41,42,43,44,45,46,47]. As a result, in the case that one LoRa-modulated signal is substantially stronger (e.g., 8 to 20 dB, depending on the SFs of the signals as discussed in [47]) than the other, the weaker one will not be decoded correctly. However, [42] reveals that the impact of imperfect orthogonality is limited when the traffic load in the network is low. Another possibility is to configure the end-devices with channel BW 250 kHz and SF. The available SFs and BW, and the corresponding physical layer peak, physical layer data rate, and sensitivity for LoRa modulation are summarized in Table 1.

### 2.2. The Network Components and Medium Access Control Layer

In LoRaWAN, the network follows the star topology with gateways conveying messages from the end-devices to the network server. The on-air transmissions between the end devices and the gateways are primarily encoded with LoRa. Further communication from the gateway to the server uses an IP-based backbone connection through a wired or a wireless interface.

**Figure 1:**
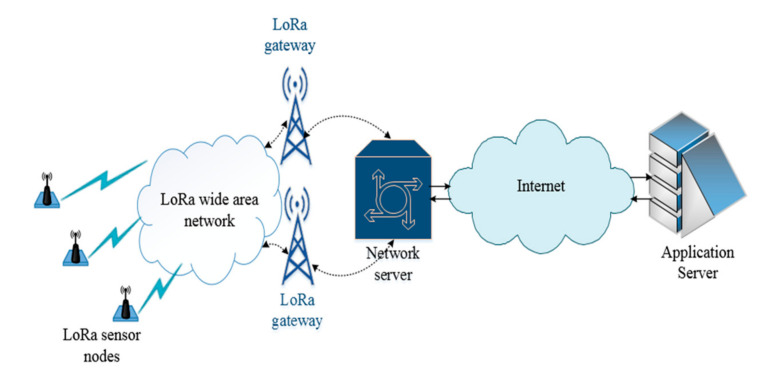
LoRaWAN network architecture.

The three key LoRaWAN network components, namely end-devices, gateway(s), and the network server, are shown in Figure 1. In LoRaWAN, class A functionality is mandatory for all the end-devices. It implies the presence of up to two receive-window-slots for acknowledgement, e.g., actuation commands after each uplink transmission. Other LoRaWAN functionalities such as class B and C are optional and imply the presence of additional receive windows. Specifically, class B enables repeated windows at certain time intervals, and the class C makes a device receive all the time it is not transmitting. These optional functionalities can enable lower latency in downlink compared to class A at the cost of higher energy consumption.

In LoRaWAN, an end-device uses ALOHA-like protocol to access the radio channel. This boosts the energy efficiency of transmission; however, the packets sent by different devices sometimes collide on-air [48]. The use of ALOHA places LoRaWAN operating in the EU under rather stringent limitations imposed by the frequency regulations for the duty cycle, which is defined as the maximum cumulative on-air time in a one-hour period. Depending on the channel and the frequency band/subband it belongs to, the maximum duty cycle permitted ranges from 0.1 to 10%. Taking a conservative requirement (i.e., 1% duty cycle), it can be calculated that a device may be on a channel for only 36 s in an hour. Note, that all three default LoRaWAN frequency channels belong to the same frequency subband (i.e., g1) and thus the 1% restriction is cumulative for these three channels. The time-on-air for the case of 24-byte packet payload under the coding rate of 4/5 (dictated by LoRaWAN specification for the EU bands) and the respective EU restrictions for the frequency channels used in our network are detailed further in Table 2.

To escape the duty cycle limitations imposed by the frequency regulations and boost the LoRaWAN data transfer reliability, which is especially relevant for the industrial use cases, the use of the listen-before talk (LBT) or carrier sense multiple access (CSMA) with LoRaWAN has been investigated [45,46,47,48,49,50,51,52,53]. To provide an example, the potential of LBT in comparison to a conventional ALOHA for LoRaWAN was explored in [46,47]. The study [47] also compared the complexity of implementing LBT at the physical layer through energy detection and at the MAC layer through frame decoding. It has been found that introduction of LBT has both positive (e.g., the improved delivery rate for dense network and the potential for increasing the throughput, especially relevant for downlink) and negative consequences (e.g., the increased energy consumption and potentially lower performance in the multigateway environment). The use of slotted ALOHA and schedule-based (i.e., time-division multiple access (TDMA)) protocols have also been suggested [51,54,55,56]. The latter ones have been found especially beneficial for industrial use cases requiring guaranteed latency and higher reliability [55,56].

Importantly, for LoRaWAN, multiple gateways can receive a packet sent by an end device. In this case, multiple copies of the same packet from the gateways reach the network server. Therefore, a LoRaWAN network server requires to filter replicas of data before storing and forwarding data to applications. In addition, a network server is responsible for ensuring data security, sending the acknowledgement, etc. Another interesting feature is that LoRaWAN technology has the capability of providing an adaptive data rate (ADR). With this optional feature enabled, the value of SF can be changed depending on the estimated communication channel condition, thus allowing a device to save energy and reducing the probability of the collisions.

## 3. Smart Campus Monitoring Planning and Deployment

**Figure 2:**
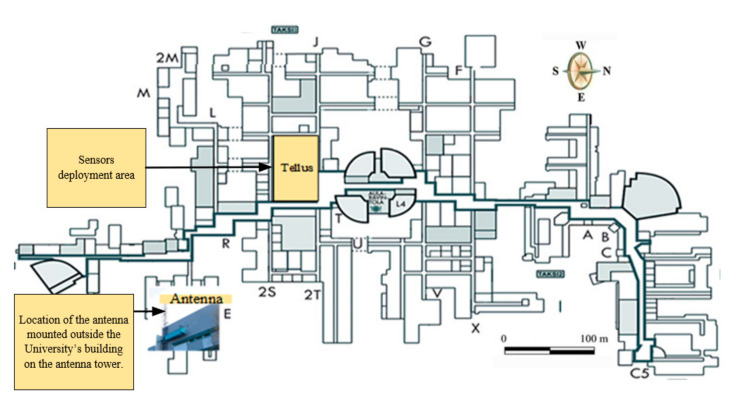
University of Oulu indoor map showing the location of the target area and the gateway.

**Figure 3:**
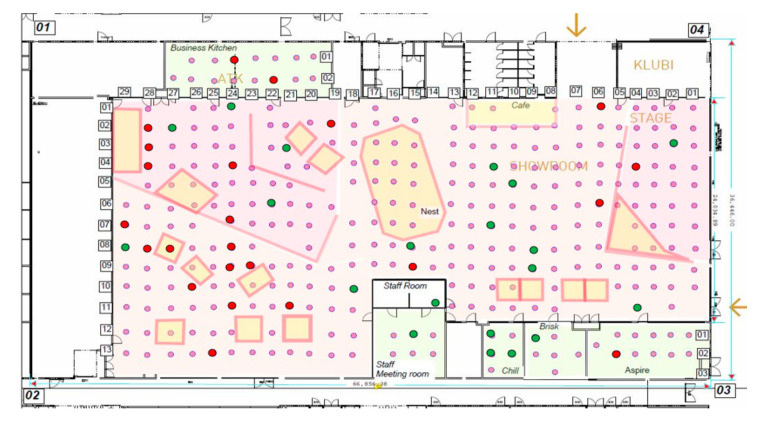
The floor plan showing the sensor node deployment in Tellus area inside the University of Oulu. The deployed sensors are shown as dots, distances are shown in meters, green dots indicate the sensors with the lowest packet error rate (PER) and the red dots are the ones with highest PER. Further details are provided in Section 4.

The earlier studies have shown that LoRaWAN technology can be an efficient solution to enable connectivity for the sensors located outdoors and scattered over significant areas. However, we hypothesize that this technology can also become a reasonable solution for indoor deployments. This has been one of the motivations for our deployment and this study. Another crucial driver for our deployment has been the introduction and the development of the Smart Campus concept as a part of the 6G Flagship program [57]. Specifically, to support both the innovation and boost the wellbeing of students and the staff, a way to collect versatile sensor data is required. Therefore, (i) in order to validate our hypothesis and understand better the suitability and limitations of LoRaWAN technology in the context of smart campus in particular, and large-scale indoor deployments in general, and (ii) enable the collection of the relevant sensor data for Smart Campus, a large-scale LoRaWAN sensor network has been deployed in the Linnanmaa campus of the University of Oulu (see Figure 2). The reason for selecting the place, i.e., the Tellus Innovation Area (Tellus) (Figure 3), was twofold. First, the whole target area is large—it stretches across 2163.8 square meters. Second, the place is mostly open and separated into different sections for brainstorming, exploring diverse cultures from inside and outside of the university, facilitating activities related to university–company collaboration, entrepreneurship, and international affairs, therefore, providing an environment where a multitude of different actions and hence services can be developed. Apart from the festivities and before the COVID-19 outbreak, the place was usually full of students and staff members during weekdays. On the one hand, this facilitates the possibilities of enabling various experimental studies. On the other hand, this brings the possibilities of improving the service quality of implemented applications based on the analyzed results from the data acquired.

To maximize the utility of the system, for our deployment, we selected the LoRaWAN-enabled sensor nodes hosting several sensors, which can measure different environmental parameters. These include the temperature, humidity, and CO_2_ levels. In addition to these, the sensor nodes are able to monitor light intensity and to detect movements. It is also worth noting that a typical passive infrared (PIR) sensor of the node can detect the movements within a range of two meters.

### 3.1. The Methodology of Deployment

In order to deploy the system, we followed the following methodology. First, we identified the positions of the sensor nodes mindful of the targeted area layout and the characteristics of the sensors (e.g., the sensing range). The total number of the sensor nodes was one of the outcomes of this planning phase. Next, we conducted a predeployment analysis to confirm the feasibility of the target deployment. In the predeployment analysis, we did an initial experiment which ensured that our target area could be covered by a single gateway located at the selected location [27]. Third, to cover the whole target area with a single gateway, we selected a place for the gateway and its antenna, as shown in Figure 2. Fourth, to select the DR/SF(s), we estimated the signal quality from several locations of the target deployment area. Once the test activities were completed, the sensor nodes were configured and deployed. Finally, the network was tested again in order to ensure that the entire setup worked properly.

### 3.2. Initial Planning

During the initial planning phase, the test area was surveyed, and the positions for the sensors selected. We decided to place our sensors on the ceiling of the area, attaching them to the rails housing the lamps. The reasoning for attaching the sensors to the rails was twofold. First, this position provides a good field of view for the sensors, which is especially crucial for the PIR sensors. Second, the sensors do not hinder mobility in the area and have a low chance of getting damaged. In addition, since the lamp rails are positioned regularly 1.5 m apart from each other, the sensors can be placed to form a regular grid. The distance between the sensors on a single rail was determined based on the sensing range of the PIR sensor and set equal to 1.5 m. Our calculations showed that to cover the whole area, about 400 sensor nodes were required. From this number, we excluded some located next to or right upon the walls and other partitions separating the sections of the deployment area, in the end having 331 sensor node positions, depicted in Figure 3.

### 3.3. Predeployment Network Analysis

In this phase, a predeployment analysis was carried out to select the configurations that can be enabled for the targeted requirements in our deployment. To understand if the proposed deployment is feasible and get an insight into the performance limits of the deployment, we have carried out some analytical work. As discussed above, the LoRaWAN can utilize different DR/SF(s) to transmit packets over the frequency channels. Thus, we estimated the expected rate of collisions that happen if more than one node attempts to send a packet at the same time on the same channel, for the different number of nodes, DR/SF settings, and the period between the reports.

Specifically, consider M sensor nodes connected to a gateway and sending the packets periodically using the same DR/SF value for the whole network. Additionally, there are C frequency channels, which a sensor node can use for transmitting its packet. The time-on-air $T_{OA}$ duration can be calculated from Equation (1). We also make the following assumptions:

- Each packet has constant length since every node has sensors measuring five parameters and data payload size are fixed for each sensor [58]; therefore, all the nodes require a fixed number of bytes to convey their measurements to the network server (NS).
- No interference from external third party networks is present. This was validated through the experimental measurements before the experiments by the measurements carried near to the desired gateway position.
- The period between sequential transmissions exceeds the duration of transmission back-off due to duty cycle restrictions, as this is required by the EU frequency regulations in force (see Table 2) [49].
- No acknowledgments, adaptive data rate, or regular downlink transmissions are present, since these are neither obligatory nor required for our application, thus implying “best-effort” delivery on the one hand, and may compromise the uplink delivery probability due to half-duplex architecture of the gateway [59].
- The start-up time for each node is random, as no specific procedure for powering them up or connecting them to the network is implied.

**Figure 4:**
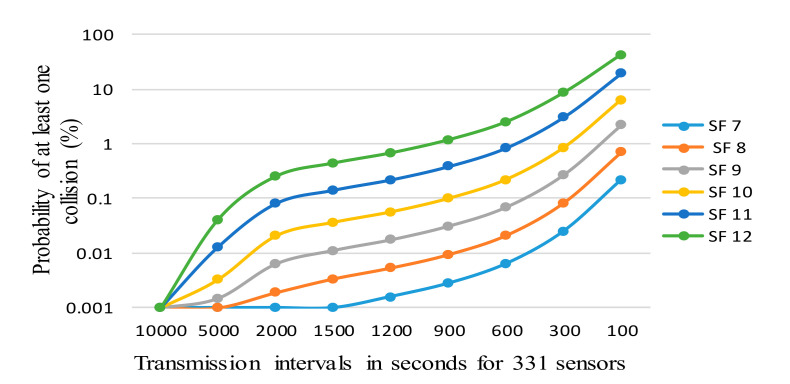
Effect of the spreading factor (SF) and report period on the probability of collisions for 331 sensor nodes (24-byte payload, C = 3 channels, one packet per interval T, calculated using Equation (2)).

We assume that the packet generation cycle is uniformly distributed over a specific time frame denoted by T. In accordance with Poisson distribution, the expected number of packets generated within the time frame T is calculated by multiplying time-on-air for each packet with arrival rate λ, which equals to M⁄((T × C)) for our case. Thus the probability of a collision is given:

(2) $$P_{k}\left(T_{OA}\right)=1-\left(\sum_{k=0}^{1}\frac{{\left(\;\lambda \times \;T_{OA}\right)}^{k}\;exp^{-\left(\;\lambda \times \;T_{OA}\right)}}{k!}\right)$$

where *k* equal to 0 and 1 corresponds to the case when either no packets or only a single packet is transmitted in a channel during the given period, respectively. The packet collision occurs when *k* > 1. The effect of the SF and T on the probability of having at least one collision is illustrated in Figure 4. The result reveals that for 15 min (=900 s) the collision probability varies from less than 0.01% to 1%, depending on the SF. This level of packet loss we consider tolerable. At the same time, a 15 min sampling period does not drain the battery too fast and enables tracking the environment parameters with a sufficient level of accuracy (as the environment parameters do not change frequently).

### 3.4. Deployed Network Elements and Configuration

For the sensor network deployment, we selected the sensor node produced by the Elsys [60]. There were several reasons for selecting this node. First, each node comprises five sensors, measuring temperature, humidity, CO_2_ level, light intensity, and movements. This sensor node is powered by two 3.6 V AA-sized lithium batteries allowing for a multiyear lifetime. Once a user configures the sensor node, all configurations get loaded from the internal memory [60]. Second, the user can configure or modify the settings by using an Android phone with near-field communication (NFC), which is embedded into the nodes. For security purposes, we used a PIN code for every node, ensuring that no unauthorized reconfiguration could be made. Third, the configuration parameters, such as the minimum, maximum, or default DR, or the transmission interval, could also be changed by a command sent in LoRaWAN downlink. Finally, the data format of the sensors was available and could be modified according to the user requirements, which enables standalone deployments.

For a LoRa gateway, we selected the Multitech [32] Conduit, which is able to connect with third party’s sensor nodes in both public and private networks. This gateway features a Node-Red graphical interface, a drag and drop interface that offers easy development methods for nonprogrammers. Both LTE and Ethernet can be used for backend. This gateway model was selected in 2017 from just several options available, also keeping in mind the potential for further extension of the network to cover the whole university campus. Therefore, we installed the gateway in the premises of the Centre for Wireless Communications, and the omnidirectional sub-GHz band antenna was mounted outside the university building on the antenna tower. Biconical D100-1000 manufactured by Aerial was the antenna used, with a 2dBi antenna gain. The height of this antenna is approximately 24 m from ground level (thus the height difference between sensors and the gateway is about 21 m) and the distance to Tellus area is about 200 m.

Both the transmit power control and the adaptive data rate mechanisms were disabled to ensure that all the sensors and the gateway were in the known state. The end devices were configured to operate with SF7 and use the transmit power of 14 dBm. In addition, the network was configured to utilize only three default EU-band LoRaWAN channels, i.e., 868.1, 868.3, and 868.5 MHz (channels 0, 1, and 2, respectively). Note that a gateway supports up to eight different frequency channels; however, we intentionally used only three to observe the behavior of the network in the worst-case scenario. The secondary reason for this configuration is the desire to save some of the channels for the future use for implementing the application requiring higher quality of service. (These can be configured to use more channels, thus implementing a sort of prioritization mechanism, missing in LoRaWAN as of today). A packet forwarder application was installed on the network server running right on the gateway to route the packets to an IoT server. All these packets were delivered to and stored by the ThingWorx IoT server by means of the MQTT protocol through a third-party commercial MQTT broker [61]. The third-party MQTT broker was used to simplify the deployment and management of the data transfer between the LoRaWAN network server and the ThingWorx IoT server. Aside from the actual sensor data, each packet contained the radio-related parameters such as the received signal strength, battery usage, radio channel, received time-stamp, etc. The users or developers are able to access the IoT server and monitor data in real-time from anywhere through the Internet. The aggregated data can also be downloaded from the server by using the application programmable interfaces (APIs) for offline in-depth analysis.

After confirming the feasibility of the target application, we carried out the tests to assess which DRs/SFs should be used. For the deployment, we decided to configure the whole network with DR 5, implying the use of SF 7. The reasons for this selection are the lower probability of collision compared to the transmissions by higher SFs, as shown in Figure 4, while covering the whole target area with this configuration is still possible. Note that the largest distance from the nodes to the place of the gateway is approximately 260 m long. In addition, the use of low SF value minimizes energy consumption. Since the target environment does not change frequently, and to increase the lifetime of the sensor nodes and reduce the collisions probability below 0.01%, we set the transmission interval of all nodes to 15 min. To increase the security by allowing the sensors to change the keys, the sensors were configured to operate using over-the-air activation.

## 4. Network Performance Evaluation

The ultimate goal of our deployment is to facilitate digitalization in real estate. In this context, the deployed network is meant to be a trial ground for prototyping and validating the new ideas, concepts, and technologies. The investigation of the performance of the network itself is a logical first step in this way. Our study was conducted in two phases. The initial phase, which lasted for two months and was reported in [29], demonstrated the validity of our approach and confirmed that an extensive real estate monitoring system with LoRaWAN was possible. However, we realized that the packet error rate (PER) was much higher than our predeployment estimations. The analysis of the data from individual sensor nodes resulted in an initial conclusion that none of the nodes lost more than 25% of their packets, while some nodes featured 0.5% PER. It is also worth noting that the average PER from all the nodes was 8.56% during these two months with no acknowledgment and retransmission mechanisms used.

Therefore, to understand the reasons behind this and to evaluate the performance of the whole network in a long-term run, an extensive analysis was performed in the second phase. The results are presented in the two followings subsections, revealing the performance of the network from October 2017 to October 2019 and from October 2019 to January 2020. The reason for dividing the results into the two sections is the change of the environment, and, specifically the disappearance of an external interference affecting the performance of the network. Therefore, the former subsection details some of the hypotheses and the results of their validation (revealing also few interesting dependences and observations), whilst the latter reveals the current-day performance of the network.

### 4.1. Network Performance from October 2017 to October 2019

In the two following subsections, we describe the performance of the network during the first two years of its operation. We start by illustrating and discussing the change of the network and individual device performance in time and then analyze the effect of the frequency bands on the network performance.

#### 4.1.1. Short- and Long-Term Temporal Fluctuations of Performance

**Figure 5:**
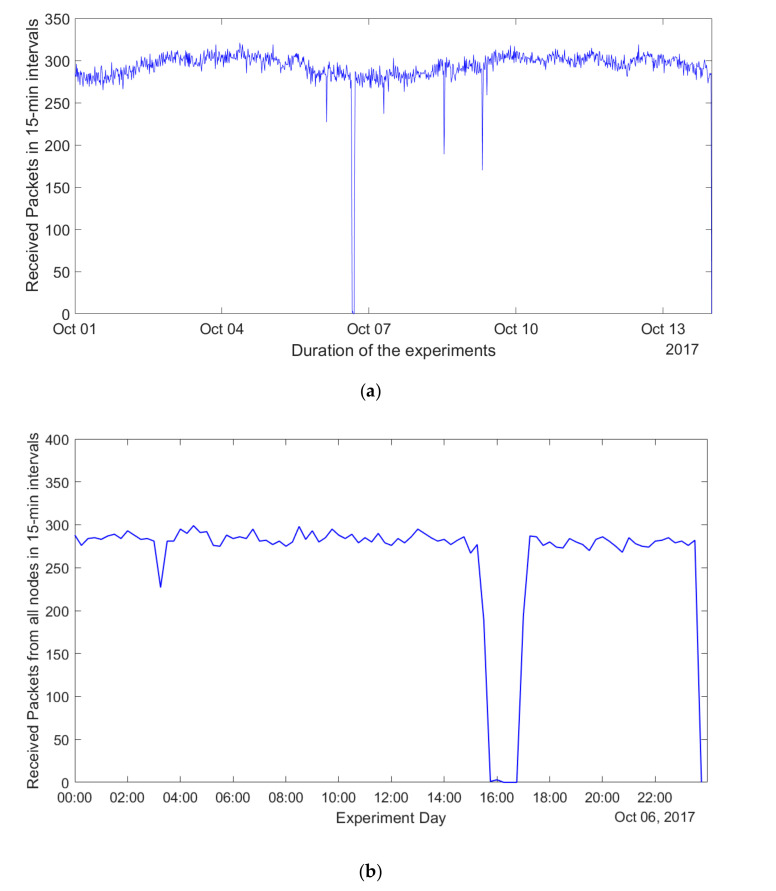
Packet reception by the IoT server. (**a**) Number of packets received in a 15-min interval from all nodes from 1 October to 14 October 2017. (**b**) Number of packets received in a 15-min interval from all nodes on 6 October 2017.

When analyzing the results, it is important to note that all 331 sensors were configured to send their packet with a period of 15 min, and we did not follow any specific pattern when starting the sensors. Therefore, if no packets are lost, 331 packets should be received within each 15-min interval. To validate this, we divided the time into 15 min intervals and calculated the number of packets received by the gateway in each of them. The illustrative results for the period from 1 October to 14 October 2017 are plotted in Figure 5a. One can see that most of the time the number of the packets received by the gateways was between 250 and 300. However, there were also several intervals when much fewer and even no packets were received. For example, during 6 October there were few consecutive intervals (i.e., 15:00 to 16:30) when no packets were received (for convenience, the total number of packets received in a 15-min interval for 6 October 2017 is illustrated in Figure 5b).

To understand the reasons for this effect, we analyzed the logs and the statistics on the number of the received packets on the gateway, as well as the sequence numbers of the individual packets recorded in our database. These show that the sensors and the gateway were operational and did not experience any resets. In addition, the logs of the IoT server show no technical problems. Importantly, no manual operations were executed before recovering from such a period characterized by high packet loss. This leads us to the conclusion that the most likely reason for such a behavior is the loss of the packets in the backbone network (i.e., between the LoRaWAN network server and the IoT data server). Since we were interested primarily in the performance of the on-air LoRaWAN network, we excluded from further analysis the periods when the issues with the backbone might have affected the performance of the network (i.e., the periods when more than 50% of the packets were lost). On average, such periods happened once every 1.5 months.

**Figure 6:**
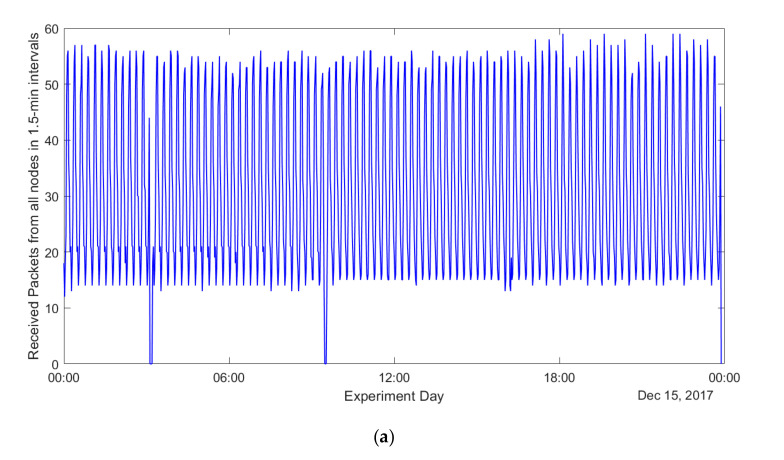

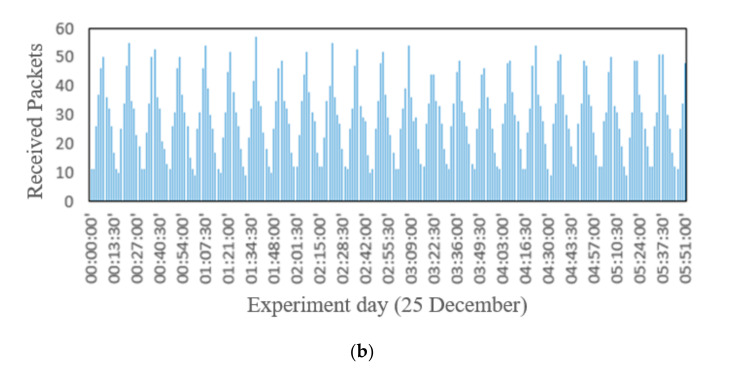
Packet reception by the IoT server on different days. (**a**) Number of packets received in a 1.5-min interval from all the nodes for a day (15 December 2017). (**b**) Number of packets received in a 1.5-min interval from all the nodes for six hours (25 December 2017).

Next, we increased the resolution to get a better insight into how the packet transmissions by the nodes were distributed within a 15-min interval. For that, the duration of the interval used for analysis was decreased ten times and set to 90 s. The results for one full day and a six-hour interval are plotted in Figure 6a,b, respectively. The figures reveal significant (from 10 to about 50 packets in one interval) and periodic (sinusoid-like) fluctuations of the number of packets received during one single interval. Given that all the sensors were started randomly, we found this result to be surprising and counterintuitive. Note that the shape of the chart may have been caused by one of the two different reasons. The former is the nonuniform transmission of the packets by the sensor nodes. The latter is the uniform transmission of the sensor nodes under the presence of a periodic interference, causing loss of packets for some of the intervals. However, the fact that during some of the intervals more than 50 packets were received (given 331 sensors and implying uniform distribution of sensor transmission in average 33.1 packets should be sent in each 90-s interval), the second hypothesis does not hold. Therefore, we conclude that the transmissions of the sensors, despite them starting randomly, are not distributed uniformly. Unfortunately, since the source code of the commercial sensor firmware used in our deployment are not available, we cannot reconstruct the logic of its behavior. One possible explanation for this is the effect of the duty cycle restrictions of the LoRaWAN gateway while operating under over-the-air activation. Specifically, consider the scenario when many sensors attempt to associate with a gateway using over-the-air activation. To successfully establish a connection, the sensor has to get from a gateway a Join Accept message in the downlink in receive window one or two in response to its connection request. Note that after transmitting such a message, the gateway will avoid using the very same frequency channel for a period of time, which is proportional to the on-air time of the packet and the duty cycle limit. Thus, if the payloads of all responses are the same (which seems logical) and the number of the sensors is huge, then the sensors will be registered in the network in batches periodically, with the periods equal to back-off durations of receive windows one and two.

**Figure 7:**
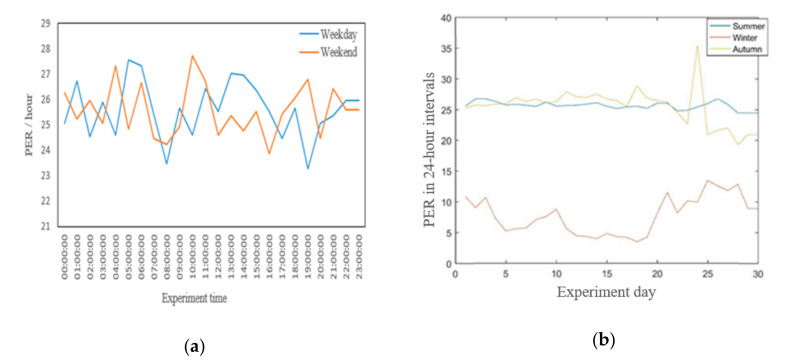
Packet error rate (PER) on the IoT server. (**a**) PER on different days for weekend and weekday. (**b**) PER on different days for three different months in three seasons.

Figure 7a,b presents the results of analysis showing the fluctuation of the network performance, namely the average PER, over longer periods of time. Specifically, Figure 7a compares the PER behavior on an hourly basis for an illustrative weekday and a weekend day. The measurement results do not reveal any notable trends—on average, 27% PER was observed for both days. Figure 7b highlights the seasonal effects on the PER. Specifically, we analyzed data collected in three separate months on three different seasons, i.e., September 2017 (autumn), December 2017 (winter) and June 2018 (summer). The results are shown in Figure 7b, where the PER in winter is less compared to autumn and summer. Only 10–15% of transmitted packets were lost in winter; however, the packets loss percentage reached 25% in autumn and summer. As noted in Section 3.4, the gateway was placed in an indoor location, but the antenna was mounted at an antenna tower outside the university building. Therefore, outdoor temperature variation and snow could have affected the communication.

#### 4.1.2. Frequency Bands and Radio Channel

**Figure 8:**
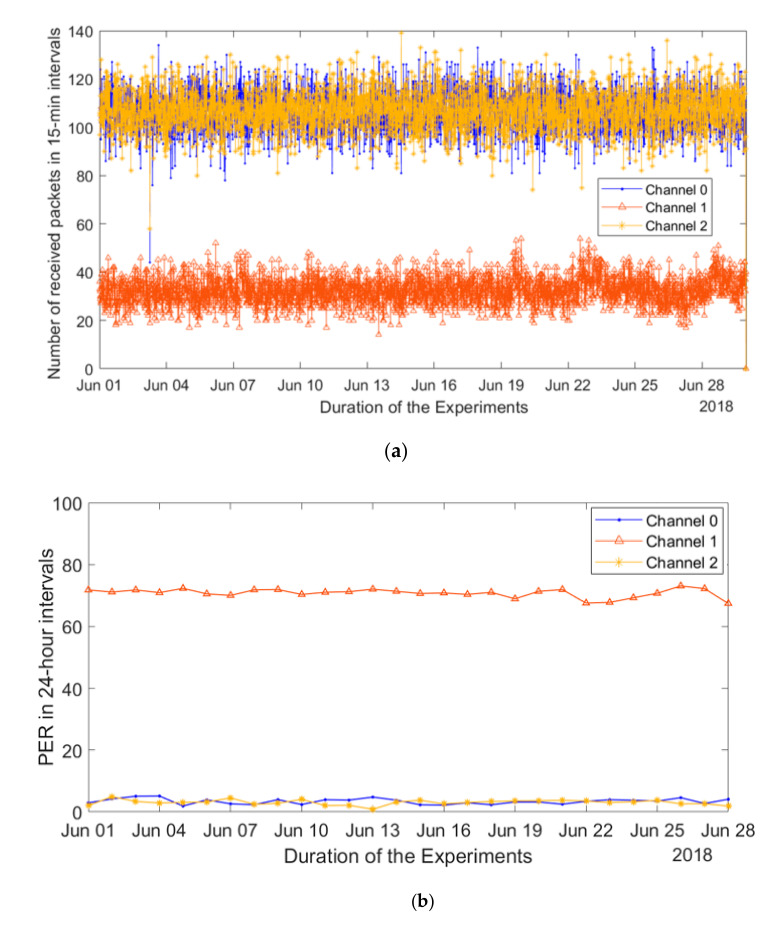
Analysis of the effect of frequency channel on packet error rate (PER). (**a**) Distribution of the received packets between the frequency channels for a month (June 2018). (**b**) Average PER for each channel through that month PER on different days for weekend and weekday.

The LoRaWAN MAC implies the use of multiple frequency channels, with three (the so-called default channels) being an absolute minimum. The channel to be used for an uplink transmission is selected by an end device randomly, keeping in mind the duty cycle restrictions. Therefore, in our further analysis, we focused on investigating whether the frequency band used affects the performance of the network. The respective results, revealing the number of packets received over each of the three channels and the per-channel PER, are depicted in Figure 8a,b. Our results reveal that the number of packets received through channel one was much (about three times) lower than that from channels two and three. Implying the uniform use of each channel (which is logical, since the duty cycle limitation for each of them is the same), in average about 110 packets should be sent per a 15-min interval in each channel. However, in channel one, only 35 packets are received by the gateway on average. Two reasons may explain this behavior, namely (i) nonuniform use of the frequency channels by the transmitters, and (ii) higher loss of packets transmitted in channel one. To get a more in-depth insight into the reason after this, we selected several nodes, which demonstrated the lowest PER throughout (2–3%) the whole duration of our measurements (sensor nodes are marked with green color in Figure 3) and investigated the distribution between the frequency channels of the packets received from these nodes. These results demonstrated a uniform distribution of the packets between the different frequency channels. Therefore, we conclude that the key reason for the observed behavior was the higher packet loss in channel one (i.e., 868.3 MHz) compared to the other frequency channels.

**Figure 9:**
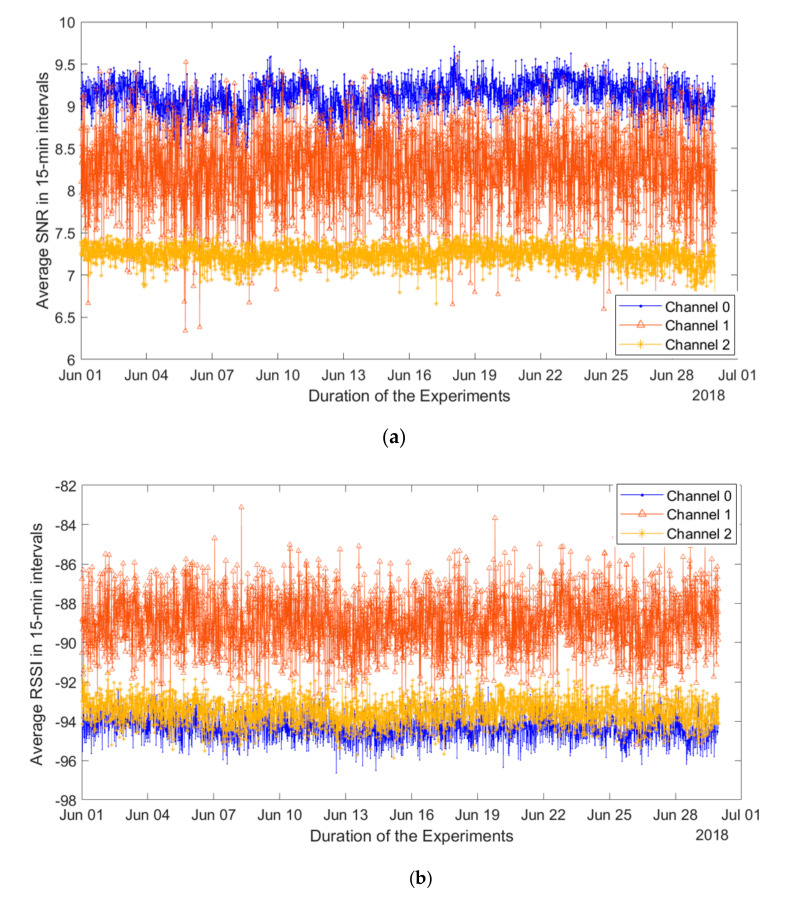
Average signal-to-noise ratio (SNR) and radio signal strength indicator (RSSI) over the channels measured by LoRaWAN gateway. (**a**) Average SNR over all the nodes in each channel with 15-min interval for June 2018. (**b**) Average RSSI over all the nodes in each channel with 15-min interval for June 2018.

We also analyzed the relevant radio channel performance metrics measured by the gateway, namely the radio signal strength indicator (RSSI) and the signal-to-noise ratio (SNR). The respective average values plotted for each frequency channel are illustrated in Figure 9a,b. From these charts, one can see that for packets in channel one, where a great number of packets were lost, the RSSI takes the highest values and SNR is the second best. This result may seem counterintuitive at first. However, it can be explained since both RSSI and SNR are measured by the gateway and recorded only for the packets, which were received correctly. The packets that were not correctly received, e.g., due to the interference, likely had low SNR and RSSI, thus not affecting the statistics. Note that the fluctuations (especially the short-term ones) for both RSSI and SNR for packets sent over channel one are higher than that for the two other channels, where packet losses were much lower. This observation may be useful for enabling detection of a potentially poorly performing channel; however, further investigation on this is required.

**Figure 10:**
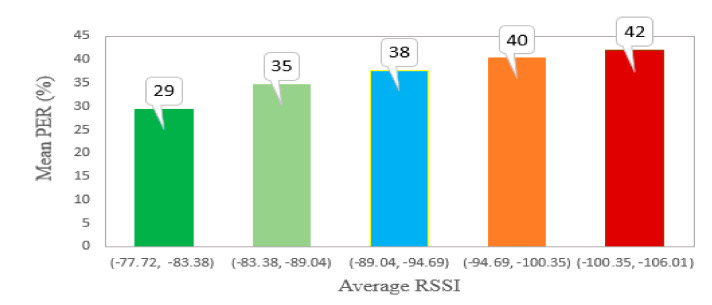
The effect of the signal-to-noise ratio and received radio signal strength on the packet error rate.

**Figure 11:**
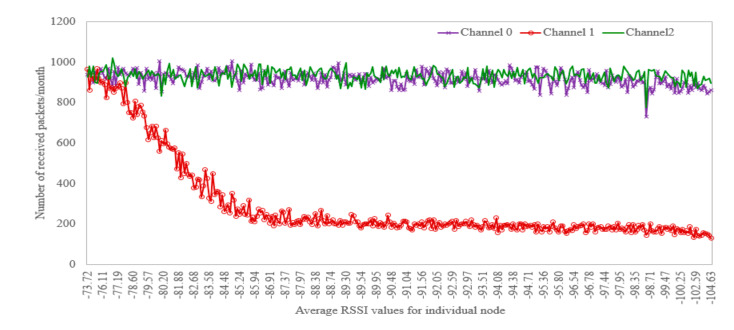
Average received radio signal strength and the number of received packets of an individual node for the different frequency channels.

We continue the analysis of the channel effect on the communication performance by investigating how RSSI affects PER. For this, we divided the whole observed range of average sensors’ RSSI values into five bins and estimated the average PER for all the sensors in each bin. The results are depicted in Figure 10. Not surprisingly, with the increase of the average RSSI, the PER increased. However, it can be seen that this increase was not very sharp. To detail this further, we also estimated the effect of the RSSI on the number of packets received in the different frequency channels. For this, we sorted all the sensor nodes based on the average RSSI of their signals and then plotted the number of the packets received from these sensors over each frequency channel. These results are depicted in Figure 11. It can be seen that for the sensors with a high RSSI (i.e., 73–77 dBm), the number of packets received over each of the three channels is about the same. However, with the increase of the channel attenuation between an end device and the gateway, a number of packets received in channel one drops greatly. When the RSSI falls below −85 dBm, the number of packets received over channel one saturates at about 20–25% of that received in the other channels.

From the results discussed above, we conclude that the key reason for the observed high packet losses is the interference from a third-party system. Note that the LoRaWAN network operates in the license-free 868 MHz ISM band, which is open to any other system, which follows the restrictions imposed by the frequency regulations (discussed in Section 2.2). Meanwhile, the fact that only one of the three default LoRaWAN channels is affected clearly shows that the interference comes not from another LoRaWAN network. To confirm this hypothesis, we also contacted the known operators of the commercial LoRaWAN networks, who also confirmed that they observe a higher PER in this particular frequency channel in their networks around Oulu city.

### 4.2. Network Today

**Figure 12:**
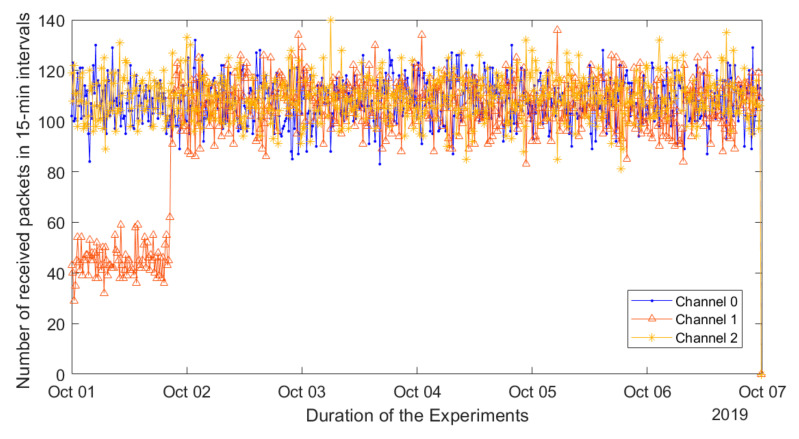
Analysis of the effect of frequency channel on packet error rate from 1 to 7 October 2019.

After concluding that the operation of the network is affected by interference from a third-party system, we attempted to localize the source of the interference. At first, we contacted the other research units and university services asking whether they were aware of any system operation in the band overlapping with channel one of the LoRaWAN network. Not getting any response, we were about to start searching for the interfering system by means of radio direction finding methods. However, it was discovered that the interference has vanished. The effect of this is illustrated in Figure 12. As one can see, on 2 October 2019 the number of packets received over channel one suddenly increased to the same level as that in the other channels. Since that time, we have not noticed any significant drops in the performance of this channel and the average PER has stayed below 1.85% for the whole network over the following half year of operation.

## 5. Discussion and Conclusions

The paper detailed the methodology of deployment and presented the results of a comprehensive analysis of the performance of a LoRaWAN-based real estate monitoring system, deployed inside the University of Oulu campus as a part of the Smart Campus development program. This program offers an open platform for research, development, and piloting novel services and solutions that can come up in future smart cities [57]. To the best of our knowledge, this paper is one of the first studies investigating such an extensive indoor LoRaWAN network (331 end devices) over this long a time (over two years). Our initial results (reported in [29]) showed substantial (about 8.56%) packet losses in the network, which motivated us to look deeper in this issue to understand the reasons for it. The analyses reported in this paper lead to several interesting findings and observations:

- It is conventionally implied that the wireless channel is the only place where the packets may get lost in an LPWAN. However, our results showed that packet losses in the IP-based backbone network (in our case composed of the pan-university local area network, the third-party commercial MQTT broker, and IoT server) also happen. Moreover, these can cause that no packets are delivered for periods of a few minutes to a few hours. This fact calls for the development of efficient solutions for detecting and managing such situations (e.g., through periodic heartbeat messages or use of packet buffering).
- Another conventional implication relative to LoRaWAN networks is that collisions primarily cause packet losses in a wireless channel. After extensive analysis of the data for over an 18-month period, we identified that the primary reason for packet losses in our network was interference from an external system, which has been affecting one of the default LoRaWAN frequency channels. Unfortunately, we were not able to track this external system since it has stopped its operation. However, our results may be significant for the other practitioners, allowing them to speed the diagnosis of their networks. This observation also calls for investigating and developing the novel solutions to detect and mitigate the interferences affecting the LoRaWAN networks.
- Next, in the data collected within our networks, we witnessed several interesting temporal effects. The most notable of these is the nonuniform distribution of the transmissions from the sensors within a single report period. In our opinion, this effect and the potential reason for it need to be explored in more detail, and this will be one of the directions for our further studies.

Overall, our results confirm that LoRaWAN technology can be rather effectively used not only outdoors, but also indoors even for rather extensive deployments, composed of hundreds of sensors. Based on our experience, even utilizing low SF values, the LoRa-modulated signals allow good network coverage and data transfer reliability to serve the needs of noncritical and loss tolerant applications. The use of low SF is also important to increase the lifetime of the nodes. With respect to this, during the two years of continues operation, there were only three nodes that stopped operating (with the voltage of the battery dropping below 2 V).

During its operation, the data collected from the deployed sensor network became the basis for several practical applications and was used in multiple student projects. This is one of the reasons why we recently started working towards enabling public open access to the sensor data generated by our system. In future, we intend to complete this and focus specifically on developing new innovative use cases on top of our deployment. In addition, we have already planned the extension of the network to cover the whole area of the campus, integration of the second gateway in the network, and introduction of the new sensor types (e.g., sound and parking sensors). We also plan to conduct similar experiments with other competing LPWAN technologies (especially the ones belonging to the cellular IoT (C-IoT) group) and investigate the suitability of these technologies to serve the emerging IoT use cases in the context of reliability, scalability, stability, and seamless connectivity.

## Figures and Tables

**Table 1:** Peak physical layer data rate for SFs and BW used by LoRaWAN in EU868 MHz ISM band.

Data Rate (DR)	Configuration Setup	Bit Rate (kb/s) ^1^	Sensitivity (dBm) ^1^
DR0	LoRa: SF12, 125 kHz	0.25	−137
DR1	LoRa: SF11, 125 kHz	0.44	−134.5
DR2	LoRa: SF10, 125 kHz	0.98	−132
DR3	LoRa: SF9, 125 kHz	1.760	−129
DR4	LoRa: SF8, 125 kHz	3.125	−126
DR5	LoRa: SF7, 125 kHz	5.470	−123
DR6	LoRa: SF7, 250 kHz	11.00	−122
DR7	FSK: 150 kHz	50.00	−122

^1^ LoRa Alliance. “LoRaWAN Specification,” version 1.1, release 2017.

**Table 2:** Time-on-air for LoRaWAN packets with different spreading factors (SFs) and the respective duty cycle restrictions on EU868 MHz ISM band (24-byte packet payload, CR 4/5, g1 frequency subband).

Configuration Setup ^1^	Bit Rate (kb/s) ^1^	Duty Cycle Restriction [30]	Time-on-Air (ms) [40]	Back-off Time, s
SF12, 125 kHz	0.25	1%	1482.75	146.79
SF11, 125 kHz	0.44	1%	823.30	81.51
SF10, 125 kHz	0.98	1%	370.69	36.70
SF9, 125 kHz	1.760	1%	205.82	20.38
SF8, 125 kHz	3.125	1%	113.15	11.20
SF7, 125 kHz	5.470	1%	61.70	6.11
SF7, 250 kHz	11.00	1%	30.85	3.05

^1^ LoRa Alliance. “LoRaWAN Specification,” version 1.1, release 2017.
